# Pulp sensibility test responses in patients with epilepsy: A matched case–control study

**DOI:** 10.1186/s12903-026-09447-4

**Published:** 2026-07-28

**Authors:** Emre Çulha, İrfan Yüksekkaya, Oğuz Burhan Çeti̇nkaya, Özge Yüksekkaya

**Affiliations:** 1 Department of Endodontics, Faculty of Dentistry, Gaziantep University, Üniversite Blv., Diş Hekimliği Fakültesi, Şehitkamil/Gaziantep, 27310 Türkiye; 2 Şehitkamil Oral and Dental Health Center, Gaziantep, Türkiye

**Keywords:** Epilepsy, Dental pulp, Electric stimulation, Thermal sensation, Pain threshold

## Abstract

**Background:**

This matched case–control study aimed to compare electric pulp testing (EPT) thresholds and cold test reaction times (CT) between patients with epilepsy and healthy controls.

**Methods:**

Sixteen systemically healthy individuals and sixteen patients diagnosed with epilepsy were included. EPT and CT using ethyl chloride were applied to the coronal third of maxillary central incisors. Data were analyzed using the Shapiro–Wilk test, Student’s *t*-test, and Mann–Whitney U test (*p* < 0.05).

**Results:**

The mean age of participants was 33.4 ± 1.8 years. EPT thresholds and CT reaction times were significantly higher in the epilepsy group compared with healthy controls (EPT (device unit = du): 6.81 ± 1.38 du vs. 3.81 ± 1.22 du; CT: 0.89 ± 0.18 s vs. 0.35 ± 0.12 s; *p* = 0.001).

**Conclusions:**

Patients with epilepsy exhibited significantly reduced pulp sensibility responses to both electric and thermal stimuli. Pulp sensibility test results should be interpreted cautiously in patients with epilepsy, as altered neurological processing may affect diagnostic accuracy.

## Background

People of all ages, genders, locations, social classes, and races are affected by epilepsy, a widespread chronic neurological illness marked by a persistent propensity to experience recurrent epileptic seizures [1]. Active epilepsy has an incidence of 61.4 per 100,000 person-years and a prevalence of 6.4 per 1,000 [2]. Antiseizure medication (ASM), surgical procedures, or stimulating the vagus nerve are the three methods used for managing epilepsy [3]. Although ASMs are well tolerated at therapeutic doses, they may have side effects, such as dizziness, fatigue, or gastrointestinal issues, which can affect patient adherence to treatment [4].

Thermal and electrical pulp sensitivity tests are employed in clinical settings to non-invasively evaluate the condition of dental pulp nerve fibers, although they are subjective and patient-dependent [5]. The most commonly used pulp sensitivity tests are the cold test (CT) and the electric pulp test (EPT), which stimulate the pulp nerves by causing the dentinal fluid to flow through temperature changes or by conducting an electric current [6].

EPT is a simple, reproducible, non-invasive technique that identifies the qualitative sensory symptoms of tooth pulp [7]. However, different clinical conditions may affect EPT results [8]. The operating principle behind EPT is to cause an ionic shift in the nerve membrane by electrical impulses that impact the action potential through fast jerking movements in the Ranvier nodes of myelinated nerves belonging to A-delta fibers located in the pulp tissue [5].

CT causes the flow of tubular fluid through temperature changes, which subsequently activates the nerve terminals found inside the dentinal tubules that function as mechanical sensors [9]. CT can be performed using dry ice, ice, and refrigerant aerosols such as tetrafluoroethane, butane, propane, isobutene, dichlorofluoromethane, and ethyl chloride [5].

Peripheral neuropathy is known to alter sensory processing, pain perception, and peripheral nerve conduction. Individuals with epilepsy may present with modified somatosensory thresholds, altered cortical pain modulation, and impaired small-fiber function, independent of ASM use [10]. Additionally, ASMs like carbamazepine, valproate, and phenytoin affect voltage-gated sodium and calcium channels, which may make peripheral nerves less excitable and raise the threshold needed to start an action potential [11, 12]. These mechanisms suggest that epilepsy and its pharmacotherapy may influence the responsiveness of pulpal nerve fibers to thermal and electrical stimuli, yet this hypothesis remains largely unexplored in the dental literature. There is insufficient information on the reliability of pulp sensibility tests in patients with epilepsy. No previous clinical studies have systematically investigated the effects of epilepsy or chronic ASM therapy on pulp sensibility test outcomes. Although several systemic conditions—such as diabetes, neuropathic disorders, or chronic pain syndromes—have been shown to alter EPT and CT responses, similar evidence for epilepsy is lacking. This absence of data is noteworthy because altered sensory thresholds could potentially lead to misinterpretations of pulp testing results in this patient population, affecting diagnostic accuracy and treatment planning. Given this background, the present study addresses an important and previously unexplored clinical question by evaluating pulp sensibility responses in individuals with epilepsy. Therefore, the aim of this study was to assess and compare EPT thresholds and CT reaction times in patients with epilepsy and healthy individuals. The null hypothesis was that neither EPT values nor CT reaction times would differ between the two groups.

## Methods

Gaziantep University Ethics Committee for Non-Interventional Clinical Research approved all of the processes carried out in this case–control study (Decision No: 2025/163, Date: 21/05/2025). Prior to the study, each participant provided written informed consent. Every technique utilized in the study was carried out in compliance with the latest version of the Declaration of Helsinki.

In the present study, the terms “sensibility” and “sensitivity” are used with distinct meanings. Pulp sensibility refers to the neural response of the dental pulp to external stimuli, typically assessed using EPT and thermal tests (e.g., CT), which primarily stimulate A-delta nerve fibers. These tests evaluate the functional status of pulpal innervation rather than true pulpal vitality. In contrast, “sensitivity” is a broader term describing the ability to perceive or detect a stimulus and is often used in a diagnostic context to indicate the performance of a test. To avoid confusion, the term “sensibility” is used throughout this study when referring to pulp testing outcomes.

The method used in the present research was identical to that employed by Owlia et al., which compared the pulp responses of uncontrolled type II diabetes patients with those of well-controlled ones [13]. An analysis was performed using G*power 3.1 in order to obtain statistically significant results at a satisfactory level. This value was derived from prior research [13–15]. For a statistically significant difference of 0.6 ± 0.5 (Cohen’s d = 1.20) units in pulp sensitivity measurements between patients with epilepsy and the (healthy) control group, the minimum number of participants required in each group was 16 (α = 0.05, 1-β = 0.90). To ensure homogeneity with regard to age and gender, 16 participants with epilepsy and 16 healthy participants without systemic illness were recruited using convenience sampling from two clinical sources. Patients with epilepsy were recruited from the Neurology Department, whereas healthy controls were recruited from the Department of Oral Radiology. The groups were frequency-matched for age and gender to improve baseline comparability.

Although epilepsy is a relatively common neurological condition, strict eligibility criteria were applied to minimize confounding factors affecting pulp sensibility responses. Participants with systemic diseases, smoking, dental trauma, restorations, caries, periodontal problems, parafunctional habits, relevant medication use, or other neurological/metabolic disorders were excluded. Therefore, the final eligible sample was limited. The calculated minimum sample size was 16 participants per group, and this requirement was met. The participants included in this descriptive cross-sectional study (8 male and 8 female in every group) were between the ages of 18 and 54. The age and gender distribution was deliberately made homogeneous across groups. Frequency matching was applied rather than individual matching. Equal numbers of male and female participants were included in each group, and the age distribution was kept comparable between groups. Baseline comparability was assessed statistically. Patients with epilepsy were clinically monitored at the neurology department of a medical faculty hospital in X. At least one year had passed since the diagnosis of epilepsy in this group. 16 healthy participants without past evidence of chronic illness were referred from X University, Faculty of Dentistry, Department of Oral Radiology, to serve as the control group. Patients were not stratified according to epilepsy subtype or antiepileptic drug regimen, as the primary objective was to evaluate the overall effect of epilepsy as a neurological condition on pulp sensibility.

The exclusion criteria included participants with maxillary central incisors presenting incomplete root formation, caries, restorations, periodontal disease, or mobility greater than Grade I; those who had used analgesics within the previous 48 h or tricyclic antidepressants or antihypertensive medications within the previous 3 months; and healthy control participants with a history of anticonvulsant use within the previous 3 months to avoid potential confounding. Participants with a history of drug dependence, previous orthodontic treatment or dental trauma, other neurological, nutritional, or metabolic disorders, cracked or suspected cracked teeth, bruxism or other parafunctional habits, fixed orthodontic retainers, or smoking were also excluded. Participants were classified as smokers if they answered “yes” to at least one of the following questions: *“Have you smoked at least 100 cigarettes in your lifetime?”* and *“Do you currently smoke?”*

To prevent any bias, all participants were assigned a code number to preserve anonymity. Thus, the evaluator did not know whether the participants were patients with epilepsy or healthy individuals. Sensitivity tests were performed by the same operator (İY) on a single maxillary central incisor in each subject. A 5-minute interval was allowed between EPT and CT to ensure that the effects of the previous test on the participants had worn off. The participant’s age, gender, and tooth number were also recorded.

All participants were informed about the procedure before the start of EPT. EPTs were performed by a single operator (İY) who had been using electric pulp test devices for at least 5 years, in accordance with the manufacturer’s recommendations. In this way, possible inconsistencies among operators regarding the correct application of the technique were prevented. The surface of the teeth was cleared of tartar and plaque before the procedure. After drying the teeth using cotton rolls and isolating them, electrocardiographic gel (BP Ultra Gel, Turkuaz Sağlık A.Ş., Türkiye) was placed on the crown’s buccal surface as a contact material. The probe of the EPT unit (Gentle-pulse, Parkell, NY, USA) was positioned on the coronal third of the tooth. The test for each tooth began when the probe touched the enamel of the tooth and ended when the participants lifted their hands to indicate that they felt the first sensation. Responses from participants were recorded as the number that indicated the pulp tester’s current state. To minimize the effect of one test on another, the measurement was repeated three times with a 2-minute delay using the same EPT unit, and the average of the results was recorded.

CT was performed using ethyl chloride spray (Walter Ritter, Hamburg, Germany) by applying from a distance of approximately 2–3 cm from the cotton pellet. The spray was administered for about 1 s to adequately saturate the cotton ball without excess dripping. The cotton pellet was then immediately placed on the mid-buccal surface of the maxillary central incisors until the participant responded within 5 s or raised their hand when they felt pain or cold. The duration of the participants’ response to the stimulus by raising their hands was measured and recorded in seconds using a digital chronometer.

### Statistical analysis

Baseline demographic characteristics (age and gender) were compared between the groups to assess group homogeneity by using the chi-square test. Prior to statistical comparisons, the distribution of continuous variables was assessed for normality using the Shapiro–Wilk test and visual inspection of histograms and Q–Q plots. For variables that followed a normal distribution, parametric tests were applied (Student’s *t*-test for between-group comparisons). For variables that did not meet normality assumptions, non-parametric tests were used (Mann–Whitney U test).

Descriptive statistics were presented as mean ± standard deviation (SD) for normally distributed data and median (interquartile range, IQR) for non-normally distributed data. A two-tailed *p* value < 0.05 was considered statistically significant. All analyses were performed using the SPSS 22.0 software (IBM Corporation, Armonk, NY, USA).

## Results

Baseline demographic characteristics were comparable between the groups, with no statistically significant differences in age or gender distribution. The overall mean age of all participants was 33.4 ± 1.8 years (range: 18–54 years). The mean age was 31.6 ± 2.3 years in the epilepsy group and 35.1 ± 1.1 years in the healthy control group, with no statistically significant difference between the groups (*p* = 0.70). Each group included eight female and eight male participants, indicating comparable gender distribution.

**Fig. 1:**
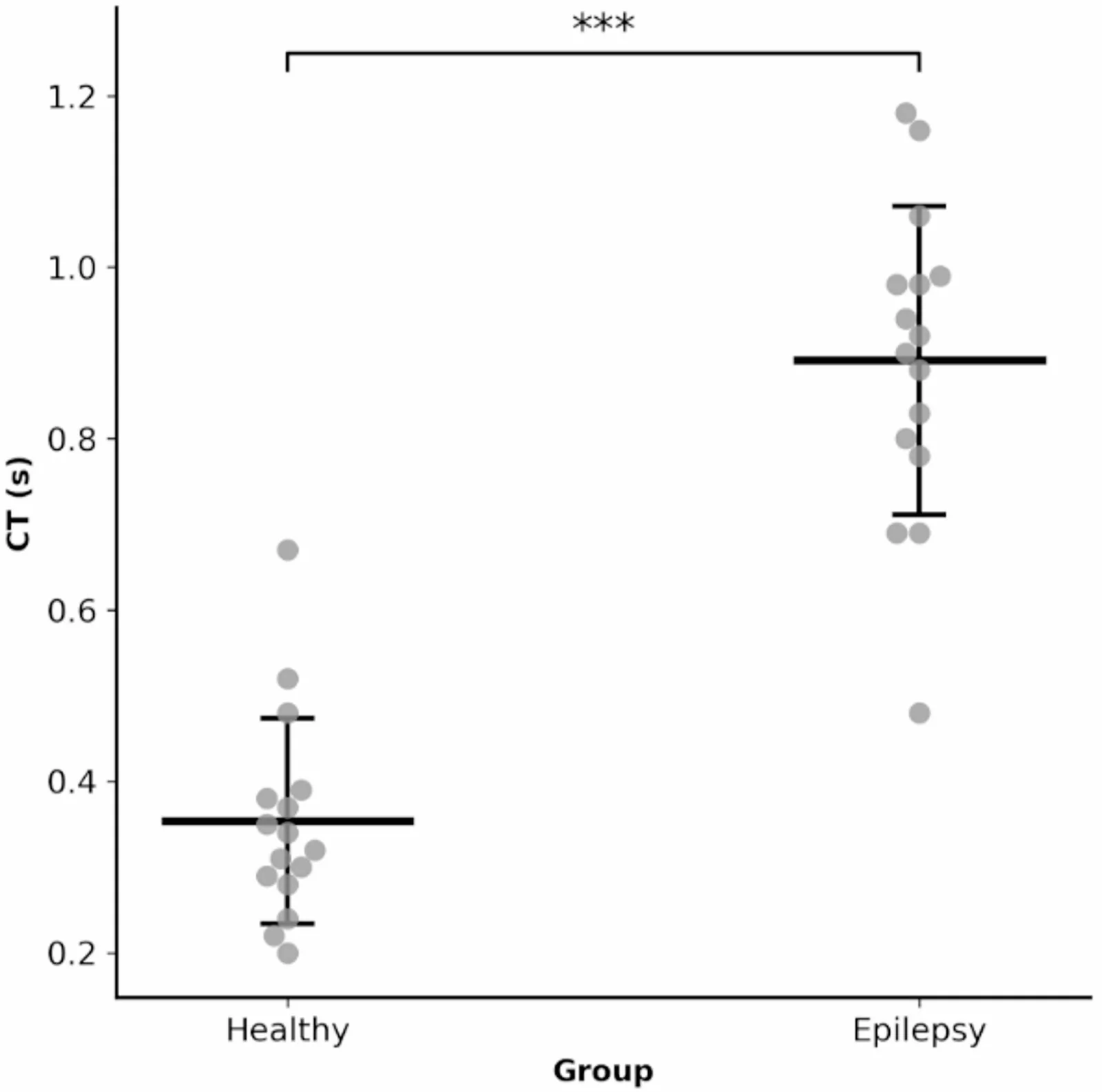
Comparison of electric pulp testing thresholds between healthy individuals and patients with epilepsy. Individual data points are represented by circles. The thick black horizontal lines indicate the mean values, and the error bars represent the SD. EPT values are expressed in digital units (du). *** indicates a statistically significant difference between the groups (*p*<0.001)

**Fig. 2:**
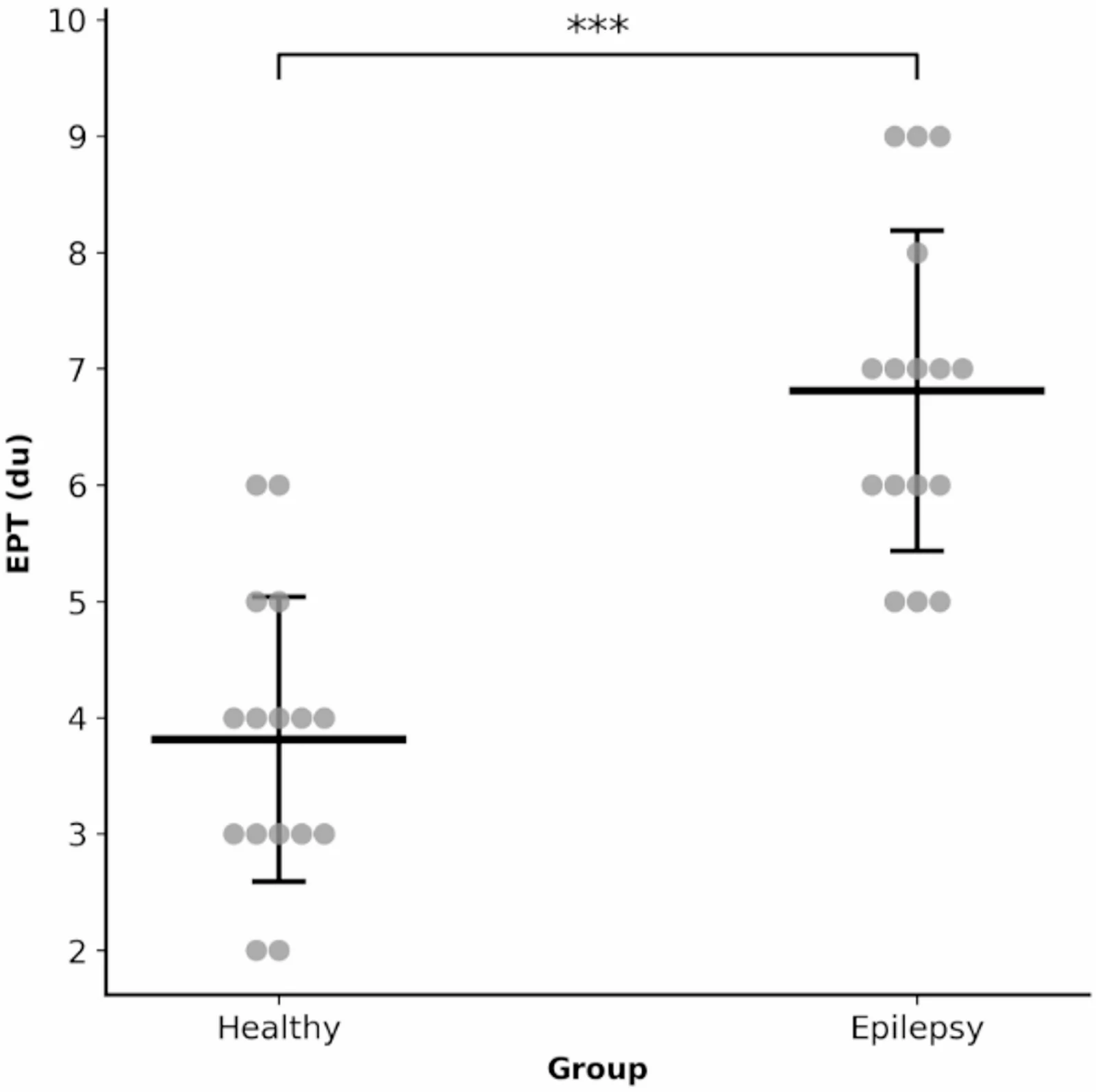
Comparison of cold test (CT) reaction times between healthy individuals and patients with epilepsy. Individual data points are represented by circles. The thick black horizontal lines indicate the mean values, and the error bars represent the SD. CT reaction times are expressed in seconds (s). *** indicates a statistically significant difference between the groups (*p* < 0.001)

Normality testing using the Shapiro–Wilk test indicated that EPT values followed a normal distribution (*p* > 0.05), whereas CT reaction times did not (*p* < 0.05). Accordingly, parametric tests were used for EPT comparisons and non-parametric tests for CT data. A difference with statistical significance was found among the EPT values and CT reaction time of the research groups (*p* < 0.05). The epilepsy group demonstrated significantly higher EPT thresholds and longer CT reaction times compared with the control group. The mean difference in EPT values was 3.00 du (95% CI: 2.05–3.95), corresponding to a large effect size (Cohen’s d = 2.30). Similarly, the mean difference in CT reaction times was 0.54 s (95% CI: 0.42–0.66), also indicating a large effect size (Cohen’s d = 3.10) (Table 1; Figs. 1 and 2).

**Table 1:** Electric pulp test values in device unit and reaction time to cold test

	Healthy participants (Control group)		Patients with epilepsy		Mean Difference (95% CI)	Cohen’s d	*P* value
	Mean ± SD	Median (Q1-Q3)	Mean ± SD	Median (Q1-Q3)			
EPT ^†^ (du)	3.81 ± 1.22	4 (3-4.5)	6.81 ± 1.38	7 (6-7.5)	3.00 (2.05–3.95)	2.30	0.001*
CT^‡^ (s)	0.35 ± 0.12	0.33 (0.29–0.39)	0.89 ± 0.18	0.91 (0.79–0.99)	0.54 (0.42–0.66)	3.10	0.001*

*EPT* Electric pulp test, *CT *Cold test, *s* Second, *du *Device unit, *SD *Standard deviation

*p < 0.05; ^†^Student’s t-test, ^‡^Mann–Whitney U test

The mean EPT threshold values (du: device unit) were 6.81 ± 1.38 du in the epilepsy group and 3.81 ± 1.22 in healthy participants. Similarly, CT reaction time was significantly higher in the epilepsy group (0.89 ± 0.18 s versus 0.35 ± 0.12 s).

## Discussion

In the present research, the reactions of dental pulp nerve fibers to EPT and CT in patients with epilepsy were compared to those of healthy people. The results revealed a statistically significant difference in pulp sensitivity among the control and epilepsy patient groups. This data suggests that patients with epilepsy may have decreased pulp sensibility. Epilepsy is primarily a central nervous system disorder and does not directly affect peripheral nerves. Observed differences cannot be directly attributed to peripheral nerve pathology and may be explained by altered central pain modulation, ASM effects, or indirect neurophysiological alterations. For this reason, our initial hypothesis that EPT threshold and CT reaction time would not change in healthy and epilepsy groups was rejected.

In order to standardize the present study, the maxillary central incisors were employed due to their ease of isolation, accessibility, lower incidence of caries, fewer contact points with adjacent teeth, and lower EPT threshold value [16]. Also, in our study, EPT and CT were performed on the incisal third of the crown rather than the cervical or middle third. This choice is supported by the studies that provide more consistent and reliable sensory recordings compared to the gingival third, where measurements taken in the incisal third may compromise test standardization due to anatomical irregularities, thinner dentine, and gingival interaction [17, 18]. To increase the calibration of participants, all patients participating in the study were selected from a single neurology centre with experienced neurologists. All patients with epilepsy included in the study had been in a controlled state for at least 1 year prior to the study.

The results of our study show that the CT reaction time in patients with epilepsy and the threshold of EPT values are significantly higher than in healthy individuals. These findings suggest that patients with epilepsy may exhibit altered responses to pulp sensibility testing; however, the underlying mechanism cannot be determined from the present cross-sectional data. Epilepsy is primarily a disorder of abnormal cortical excitability. Therefore, the present findings should not be interpreted as evidence that epilepsy directly impairs peripheral nerve conduction. EPT and CT tests assess the functions of the A-delta and C-type nerve fibers that primarily innervate the dental pulp. A-delta fibers respond to sudden stimuli with rapid and sharp pain, which forms the basis of these tests. One possible explanation is altered sensory processing or reduced neural responsiveness, but this remains hypothetical and requires confirmation in studies including neurophysiological assessments.

ASMs may represent a potential unmeasured factor influencing pulp sensibility responses. Different ASM classes have different mechanisms of action, and this pharmacological heterogeneity may contribute to variability in sensory responses [4, 19–21]. However, because detailed data on ASM type, dosage, treatment duration, and mono- or polytherapy were not collected, the present study cannot determine whether the observed differences were related to epilepsy itself, ASM exposure, or other unmeasured neurological factors. Epilepsy patients frequently undergo ASM treatment for extended periods. As a result, because most ASMs also have analgesic properties, it can be challenging to determine whether the absence of pain in epileptic patients is due to their epileptic condition or medication therapy. Since EPT and CT primarily stimulate A-delta fibers within the dental pulp, any pharmacological reduction in nerve excitability may result in delayed or attenuated sensory responses. The increased EPT thresholds and prolonged CT reaction times may reflect epilepsy-related factors, ASM exposure, or other unmeasured variables; therefore, these findings should be interpreted as associative rather than mechanistic. Due to the lack of detailed data regarding ASM type, dosage, and duration in the present study, the specific contribution of individual medications could not be determined and should be addressed in future research.

The integrity of the pulp nerve fibers and vascular structure is critical in terms of pulp vitality and the reliability of thermal or electrical pulp tests. However, these tests only evaluate the nerve response; they do not provide direct information about the actual histological condition or vascular status of the pulp [22]. Homeostatic maintenance is essential for pulp function. Disruption of pulp homeostasis can lead to pulp degeneration [23]. Microorganism invasion associated with dental caries and advanced periodontal/apical infections can lead to loss of homeostasis [24, 25]. Epilepsy patients frequently undergo ASM treatment for extended periods, dental trauma can cause sensitivity changes, ischemia or necrosis by directly disrupting the pulp blood flow and nerve structure [24].

It is important to distinguish epilepsy from other neurological and systemic conditions, such as diabetes mellitus or multiple sclerosis, that are known to directly affect peripheral nerve function and, consequently, pulp sensibility test outcomes [26, 27]. Epilepsy is primarily characterized by abnormal cortical excitability rather than structural damage to peripheral nerves. Consequently, in contrast to diabetes—where modified pulp sensitivity is predominantly ascribed to peripheral neuropathy—the alterations noted in individuals with epilepsy are more likely associated with central modulation of sensory processing or the impact of ASMs. This distinction is clinically important, as it suggests that increased pulp sensibility thresholds in patients with epilepsy should not be directly interpreted as evidence of peripheral nerve dysfunction. These pathophysiological mechanisms should be considered when interpreting the results of pulp tests performed on individuals with epilepsy. Given that systemic dosages of various forms of analgesics may alter EPT results, participants were surveyed about all types of drug usage prior to the study. Therefore, recent use of analgesics and medications that could alter sensory responses was considered an exclusion criterion.

From a clinical perspective, the findings of the present study may have important implications for endodontic diagnosis. Pulp sensibility tests are widely used to assess pulpal status; however, they rely on neural responses rather than direct evaluation of pulpal vitality. The significantly higher thresholds and delayed responses observed in patients with epilepsy suggest that these tests may yield false-negative or underestimated responses in this population. In other words, a tooth with a vital pulp may respond weakly or not at all to standard sensibility testing, potentially leading to misinterpretation of pulpal status. This has direct clinical relevance, as inaccurate test results may increase the risk of inappropriate treatment decisions, such as unnecessary endodontic intervention or misdiagnosis of pulpal necrosis. Therefore, clinicians should interpret pulp sensibility test results in patients with epilepsy with caution and consider adjunctive diagnostic methods, including repeated testing, comparison with control teeth, and, where available, more objective vitality assessments such as pulp blood flow–based techniques. However, since the present findings indicate associations rather than causal relationships, clinicians should interpret these clinical implications cautiously and validate them in larger, well-controlled studies.

The current study should be viewed in the context of its limitations. First of all, we had no detailed information regarding ASM use, including the type of medication, duration of therapy, and whether patients were receiving monotherapy or polytherapy. Given that different ASMs may have distinct effects on peripheral nerve function and nociceptive processing, the inability to stratify patients accordingly represents a potential source of confounding. Therefore, the specific contribution of individual antiseizure medications to the observed alterations in pulp sensibility could not be determined. Secondly, there was no data on how long the patients had been suffering from epilepsy. Thirdly, since the epilepsy medications used by the patients varied widely, no standardization could be achieved in this regard. Furthermore, the absence of a third comparison group consisting of patients with epilepsy who were not receiving ASMs also limits the ability to separate the potential effects of epilepsy from those of medication exposure. Also, seizure type, seizure frequency, duration of epilepsy, ASM type, dosage, treatment duration, and mono- or polytherapy status were not systematically recorded. This represents an important limitation because these variables may influence sensory processing and may act as confounders. Additionally, our study may have limited generalizability because it evaluated only a single type of tooth and was conducted at a single center. Finally, the present study’s limited sample size and cross-sectional design necessitate careful evaluation of the results.

The selection of the control group from patients attending the dental radiology department warrants careful consideration. This approach was primarily adopted to ensure the availability of systemically healthy individuals who required dental evaluations and could be examined under standardized clinical conditions, thereby facilitating consistent application of pulp sensibility tests. In addition, recruiting controls from the same clinical setting as the study group helped minimize variability related to examination procedures and operator-related factors. However, this sampling strategy may introduce selection bias, particularly Berkson’s bias, as individuals presenting to a healthcare facility may not be fully representative of the general population. Patients attending a dental radiology unit may have underlying dental concerns or health-seeking behaviors that differ from community-based individuals, which could potentially influence pulp sensibility responses. Therefore, the comparability between groups may be partially affected by the clinical setting rather than solely by the presence or absence of epilepsy. Given these considerations, the findings of the present study should be interpreted with caution. Future studies employing community-based sampling or matched population controls would be valuable to reduce selection bias and improve the generalizability of the results.

Research specific to dental pulp sensitization is limited in the existing literature. This study contributes to filling the gap in this field. The findings of the present study demonstrate that patients with epilepsy exhibit higher EPT thresholds and prolonged CT reaction times compared with healthy individuals. However, these results should be interpreted with caution. Given the observational and cross-sectional nature of the study, the observed differences represent associations rather than causal relationships. Although several potential mechanisms—such as central sensory modulation, antiseizure medication effects, and altered neuronal excitability—may plausibly contribute to these findings, the current data do not allow determination of the relative contribution of these factors. Therefore, the proposed mechanisms should be considered hypothetical and explanatory, rather than definitive. Future studies should aim to stratify patients according to ASM type, duration of use, and treatment regimen in order to better elucidate the relative contributions of pharmacological and disease-related factors to pulp sensibility responses. Additionally, incorporating objective neurophysiological assessments of peripheral nerve function may help clarify the underlying mechanisms.

## Conclusions

Within the limitations of this study, patients with epilepsy were found to have significantly higher thresholds in electric and thermal pulp sensibility tests compared with healthy controls. These findings suggest a potential alteration in sensory response; however, they should be interpreted as associative observations rather than evidence of a direct effect of epilepsy on peripheral nerve function. Clinicians should be aware that responses to pulp sensibility tests in patients with epilepsy may differ from those of healthy individuals, and diagnostic decisions should be made with caution. Further well-designed studies are required to determine the underlying mechanisms and clinical implications of these findings.

## Data Availability

The datasets used and/or analysed during the current study are available from the corresponding author on reasonable request.

## Abbreviations

ASM: Antiseizure medication
CT: Cold test
du: Device unit
EPT: Electric pulp test
s: Second
SD: Standard deviation
